# Post-COVID Quality of Life and Sleep Among Younger Healthcare Workers of Designated COVID Care Centers: A Cross-Sectional Study

**DOI:** 10.7759/cureus.38190

**Published:** 2023-04-27

**Authors:** Pradeep TS, Athish KK, Sravani Bhavanam, Bejoi Mathew

**Affiliations:** 1 Community Medicine, Sri Devaraj Urs Medical College, Sri Devaraj Urs Academy of Higher Education and Research, Kolar, IND; 2 Dermatology, Venereology, and Leprosy, Sri Devaraj Urs Academy of Higher Education and Research, Kolar, IND; 3 Internal Medicine, Sri Devaraj Urs Medical College, Sri Devaraj Urs Academy of Higher Education and Research, Kolar, IND; 4 Preventive Medicine, Sri Devaraj Urs Medical College, Sri Devaraj Urs Academy of Higher Education and Research, Kolar, IND

**Keywords:** covid-19, healthcare workers, frontline healthcare workers, covid care center, post covid, quality of life, sleep

## Abstract

Introduction

Frontline healthcare workers (FLHCWs) have been persuaded to work this coronavirus disease (COVID) pandemic way in and out but the pandemic has not subsided. The persistence of symptoms after COVID infection, especially chest symptoms like early fatigue with breathlessness, has been documented very well. However, FLHCWs have repeatedly caught the COVID infection and have been working in traumatic and helpless situations since the pandemic started. Post-COVID infection, quality of life (QOL) and sleep are greatly affected, regardless of the time elapsed since discharge or recovery. The continuous assessment of COVID-infected persons for post-COVID sequelae is an important and effective step to reduce complications.

Materials and methods

This was a cross-sectional study conducted for a period of one year at R.L. Jalappa Hospital and Research Center, Kolar, and SNR District Hospital, Kolar, which were designated COVID care centers. FLHCWs working in these centers who had contracted COVID infection at least once, who were more than 18 years and less than 30 years, and who had experience of less than five years irrespective of their vaccination status were included in the study. FLHCWs with COVID-related health illnesses, which needed ICU admission and prolonged hospital admission, were excluded. To assess QOL, the WHO Quality of Life Brief Version (WHOQOL-BREF) questionnaire was used. To assess sleepiness, the Epworth daytime sleepiness scale was used. The study was started after the institutional ethical committee's clearance was obtained.

Results

A total of 201 healthcare workers (HCWs) completed the survey. Of the participants, 119 (59.2%) were male, 107 (53.2%) were junior residents, 134 (66.7%) were unmarried, and 171 (85.1%) said they followed regular shifts. Male HCWs had higher scores in the psychological, social relationship, and environmental domains of QoL. Consultants had higher scores in all domains of QoL. Married HCWs had higher scores in the physical, psychological, and social relationship domains of QoL. Out of 201 FLHCWs, 67 (33.3%) had moderate excessive daytime sleep, and 25 (12.4%) had severe excessive daytime sleep. Few factors like gender, occupation, duration of work in the hospital, and regular shifts were statistically significant factors associated with daytime sleepiness.

Conclusion

The present study has shown that even after the COVID vaccination doses received by the HCWs, sleep and Qol have still been impaired among infected younger healthcare staff. Acceptable and righteous efforts must be made by the institutions for proper policymaking to manage such infectious outbreaks in the future.

## Introduction

Since the outbreak of coronavirus disease 2019 (COVID-19) in Wuhan, China, the world started witnessing a pandemic. The COVID-19 waves have fragmented the world of health care. Frontline healthcare workers (FLHCWs) have been forced to work this pandemic way in and out but the pandemic has not subsided [1]. Even in non-hospitalized workers, COVID has affected the health of the victims, and its post-sequelae has been less studied. Persistence of symptoms post-COVID, especially chest symptoms like early fatigue with breathlessness, has been documented very well [2,3]. A study done by Lee et al. in China has shown that in re-confirmed cases of COVID-19, physical symptoms after quarantine persisted along with feelings of depression and psychological stress. These can have a significant effect on the health of infected individuals [4]. A study done by Wu et al. has also shown that all symptoms that are caught after getting infected with COVID do not subside and require repeated consultations with the various specialties and treatment modalities [5]. Long-duration COVID symptoms can persist for one to three months after recovery even from a single infection leading to long absenteeism hampering productivity and quality of life (QoL) significantly. However, FLHCWs have repeatedly caught the COVID infection and have been working in traumatic and helpless situations since the pandemic started. The post-COVID syndrome can also have an adverse effect on the mental health of an individual. Post-COVID complications can be severe, leading to hospitalization and disability [6].

Post-COVID infection, QoL and sleep are greatly affected, regardless of the time elapsed since discharge or recovery. The ongoing assessment of COVID-infected persons for post-COVID sequelae is an important and effective step to reduce complications. There have been enormous consequences on the health of FLHCWs as they have worked relentlessly, sometimes without scheduled breaks, extended isolation, and quarantine post-exposure to suspect cases, with very less family time after exposure to the novel virus even in the era of post-vaccination for COVID [7,8]. Owing to the ongoing COVID-19 pandemic, it is a reality that the world has started to live with the novel coronavirus, and hence, it is imperative to examine the QoL to facilitate the rehabilitation of healthcare workers (HCWs), which most health institutions lack. The present COVID pandemic has also put the healthcare staff in a tricky situation where aged, well-experienced, and veteran healthcare staffs were auxiliary healthcare staff, and younger staff with immature stress management, little positive attitude toward the pandemic illness, and poor communication skills were FLHCWs working in COVID infective wards, intensive care units (ICU), isolation wards, and screening outpatient departments [9]. A study done in Japan among newer and younger HCWs working in COVID hospitals showed that younger health workers were at higher risk for developing adverse health outcomes, especially related to their mental health status, and a possible reason explained for this is fear of catching infection as they are at a greater risk being a frontline worker, with relative inexperience in treating contagious diseases, economic concerns, and poor pay during pandemic followed by not much psychological support from the social circle [10]. A study done in Canada among fresher HCWs recruited during the pandemic for COVID care showed that they required immense attention as there was a transition from academic performances to real-time life scenarios [11]. A study done in Korea showed that the younger health workforce was at higher risk of mental illness and work-related burnout because the younger HCWs were less experienced, had little training, and the unfamiliarity of their tasks increased their stress; younger individuals tend to be more involved in leisure activities and private social gatherings than their older counterparts, and their burnout may naturally be aggravated due to the restrictions imposed on these activities due to the COVID-19 infection [12]. Sleep is very essential for a better QoL, and HCWs were denied this during the pandemic. A study done in the USA among HCWs showed that three out of four HCWs had insomnia symptoms during the pandemic, which can directly affect their psychological well-being. Mental health stress can get aggravated because of poor sleep hygiene and impairs their QoL [13]. Adequate sleep and reasonable stress relief are considered indispensable elements of health, general well-being, and proper daily functioning. Insomnia might bring down clinicians' QoL by leading to cognitive dysfunction, physical discomfort, and job burnout [14].

With this background, the study was started with the objective to assess the QoL and sleepiness among COVID-infected younger HCWs working in designated COVID care centers.

## Materials and methods

This present study was a hospital-based cross-sectional study conducted at R.L. Jalappa Hospital and Research Center, Kolar, and SNR District Hospital, Kolar, which were designated COVID care centers since the start of the COVID pandemic. Both these centers covered a population of more than five lakhs. The study was done for a period of one year from January 2022 to December 2022. FLHCWs working in these centers who had contracted COVID infection at least once, who were more than 18 years and less than 30 years, and who had experienced less than five years of healthcare service irrespective of their vaccination status were included in the study. FLHCWs with COVID-related health illness which needed ICU admission, prolonged intubation, or any established complications of COVID like mucormycosis, manifestations of end-organ damage like acute respiratory distress syndrome (ARDS), cardiac injuries (ventricular arrhythmias and hemodynamic instability), thrombotic manifestations, and renal, hepatic, and gastrointestinal damage were excluded from the study. The sample size was calculated by OpenEpi (free online sample size calculation software). Sample size calculation considering the expected standard deviation (SD) of the QoL score in the FLHCWs as 25.4 and a tolerable error of 5% at a 95% confidence interval, the minimum sample size came out to be 100 [13]. For socio-demographic details, a pretested semi-structured questionnaire was used [15].

To assess QoL among younger HCWs, the WHO Quality of Life Brief Version (WHOQOL-BREF) questionnaire was used. The scale contains 26 items related to the different aspects of QoL. The scale provides a score in four different domains of QoL, namely, physical, psychological, social, and environmental. The physical domain looks for pain, discomfort, energy, and fatigue. The psychological domain looks for positive feelings, thinking and learning, self-esteem, body image, and negative feelings. The social domain looks for social relationships, social support, and social living. The environment domain looks for physical safety, security, home, and social care, along with financial resources. Each item of the WHOQOL-BREF is scored from 1 to 5 on a response scale, which is stipulated as a five-point ordinal scale [16].

To assess sleepiness, the Epworth daytime sleepiness scale (ESS) was used. All ESS item scores are intended to be integers (0-3). Scores ranging from 0 to 5 indicate lower normal daytime sleepiness, 6-10 indicate higher normal daytime sleepiness, 11-12 indicate mild excessive daytime sleepiness, 13-15 indicate moderate excessive daytime sleepiness, and 16-24 indicate severe excessive daytime sleepiness [17]. A cut-off of more than 10 is considered daytime sleepiness [18].

A list of younger FLHCWs fitting our inclusion and exclusion criteria from both COVID care centers was prepared. A total of 285 HCWs fitted the criteria out of which 201 completed the survey, which lasted not more than 20 minutes.

Ethical considerations

Informed written consent was taken from the study participants by informing them of the benefits and risks involved in the study. Study participants' autonomy and confidentiality were maintained. The study was started after obtaining clearance from the Institutional Ethics Committee, Sri Devaraj Urs Medical College (approval no.: DMC/KLR/IEC/406/2022-23).

Statistical analysis

All data were coded and entered into Microsoft Excel (Microsoft, Redmond, WA) and analyzed using SPSS version 22 (IBM Corp., Armonk, NY). After testing for normality of data by Shapiro-Wilk test and Q-Q plot, descriptive statistics like percentage, mean, and standard deviation were applied. Inferential statistical tests like the chi-square test were applied to find out the association between various factors. Student's t-test and ANOVA with post hoc Bonferroni corrections were used to compare between groups. The difference and association were expressed as statistically significant at a p-value less than 0.05. Regression analysis was done to identify the factors.

## Results

Out of 201 FLHCWs, 119 (59.2%) were male, 107 (53.2%) were junior residents, 134 (66.7%) were unmarried, 96 (47.8%) had contracted infection only once, 114 (56.7%) had received the second dose of COVID vaccination, 56 (27.9%) had been hospitalized because of COVID infection despite vaccination, 136 (67.7%) worked in COVID wards/COVID ICU more than eight hours, 171 (85.1%) follow regular shifts, 27 (13.4%) smoke cigarette daily, and 28 (13.9%) consumed alcohol once in a week (Table 1).

**Table 1:** Distribution of healthcare workers according to clinico-social factors

Clinico-social factors		Frequency	Percent
Gender	Male	119	59.2
	Female	82	40.8
Occupation	Junior Residents	107	53.2
	Consultants	25	12.4
	Nursing staff	69	34.3
Marital status	Married	67	33.3
	Unmarried	134	66.7
How many times did you contract COVID?	Only once	96	47.8
	Twice	36	17.9
	More than 2 times	69	34.3
Vaccination status	First dose	4	2.0
	Second dose	114	56.7
	Precautionary dose	83	41.3
Hours of work in the hospital	Less than 8 hours	65	32.3
	More than 8 hours	136	67.7
Regular shifts followed	Yes	171	85.1
	No	30	14.9
Smoke cigarette daily	Yes	27	13.4
	No	174	86.6
Consume alcohol weekly	Yes	28	13.9
	No	173	86.1
Admitted to the hospital for COVID-19 infection?	Yes	56	27.9
	No	145	72.1

Male HCWs had higher scores in the psychological, social relationship, and environment domains of QoL compared with female HCWs, and this difference was statistically significant. Consultants had higher scores in all domains of QoL compared with junior residents and nursing staff, and this difference was statistically significant. Married HCWs had higher scores in the physical, psychological, and social relationship domain of QoL compared with unmarried HCWs, and this difference was statistically significant. HCWs who followed regular shifts had higher scores in the physical health and environmental domain of QoL compared with those who did not follow regular shifts, and this difference was statistically significant. HCWs who were admitted to the hospital had higher scores in the environmental domain of QoL, and these differences were statistically significant (Table 2).

**Table 2:** Comparison between various groups according to different domains of quality of life

		Physical health	Psychological health	Social relationship	Environment
Gender	Male	23.7 ± 3.2	20.7 ± 2.9	20.7 ± 2.9	29.0 ± 4.2
	Female	23.5 ± 3.5	19.7 ± 2.6	19.7 ± 2.6	27.3 ± 4.0
	p-value	0.6	0.014	0.014	0.007
Occupation	Junior residents	22.3 ± 3.6	19.8 ± 3.2	19.8 ± 3.2	27.4 ± 4.9
	Consultants	25.6 ± 2.3	22.1 ± 2.0	22.1 ± 2.0	31.2 ± 2.6
	Nursing staff	25.0 ± 2.1	20.5 ± 2.0	20.5 ± 2.0	28.1 ± 2.7
	p-value	0.001	0.001	0.001	0.001
Marital status	Married	24.8 ± 3.1	21.2 ± 2.4	21.2 ± 2.4	28.8 ± 4.0
	Unmarried/separated/divorced	23.0 ± 3.3	19.9 ± 2.9	19.9 ± 2.9	28.0 ± 4.3
	p-value	0.01	0.01	0.01	0.24
Contracted COVID infection	Once	24.3 ± 3.2	20.7 ± 2.5	20.7 ± 2.5	28.9 ± 3.7
	Twice	22.6 ± 3.6	20.3 ± 3.2	20.2 ± 3.2	28.0 ± 5.4
	More than twice	23.2 ± 3.2	19.7 ± 2.9	19.7 ± 2.9	27.6 ± 4.1
	p-value	0.01	0.6	0.8	0.2
Vaccination status	First dose	21.5 ± 3.8	19.2 ± 1.2	19.2 ±1.2	25.5 ± 1.2
	Second dose	23.8 ± 3.3	20.1 ± 2.9	20.1 ± 2.9	28.1 ± 3.9
	Precautionary dose	23.5 ± 3.4	20.7 ± 2.7	20.7 ± 2.7	28.7 ± 4.7
	p-value	0.3	0.2	0.4	0.6
Are your shifts regular?	Yes	23.9 ± 3.3	20.4 ± 2.9	20.4 ± 2.9	28.7 ± 4.0
	No	22.0 ± 3.2	20.0 ± 2.3	20.0 ± 2.3	26.1 ± 4.7
	p-value	0.003	0.6	0.9	0.002
Smoke cigarette	Yes	23.5 ± 3.3	20.2 ± 3.0	20.2 ± 3.0	27.9 ± 5.1
	No	23.7 ± 3.3	20.3 ± 2.8	20.3 ± 2.8	28.3 ± 4.1
	p-value	0.8	0.8	0.8	0.8
Consume alcohol once a week	Yes	23.5 ± 4.2	20.6 ± 3.5	20.6 ± 3.5	29.3 ± 4.8
	No	23.7 ± 3.2	20.3 ± 2.7	20.3 ± 2.7	28.1 ± 4.1
	p-value	0.7	0.6	0.5	0.8
Have you ever been admitted to the hospital for COVID-19 infection?	Yes	23.2 ± 3.4	20.2 ± 3.0	20.2 ± 3.0	27.1 ± 4.2
	No	23.8 ± 3.3	20.4 ± 2.7	20.4 ± 2.7	28.8 ± 4.1
	p-value	0.2	0.6	0.6	0.01

Out of 201 HCWs, 67 (33.3%) had moderate excessive daytime sleepiness, and 25 (12.4%) had severe excessive daytime sleepiness (Table 3).

**Table 3:** Distribution of healthcare workers according to the Epworth daytime sleepiness scale

Epworth daytime sleepiness scale	Frequency	Percent
Lower normal daytime sleep	13	6.5
Higher normal daytime sleep	56	27.9
Mild excessive daytime sleep	40	19.9
Moderate excessive daytime sleep	67	33.3
Severe excessive daytime sleep	25	12.4
Total	201	100.0

Of those who had daytime sleepiness, 61 (74.4%) were female, and this association between gender and daytime sleepiness was statistically significant. Of those who had daytime sleepiness, 57 (82.6%) were nursing staff, and this association between HCWs' occupation and daytime sleepiness was statistically significant. Of FLHCWs who had daytime sleepiness, 59 (90.8%) worked less than eight hours a day, and this association between duration of work and daytime sleepiness was statistically significant. Of those who had daytime sleepiness, 117 (68.4%) followed regular shifts, and this association between regularity of shifts and daytime sleepiness was statistically significant (Table 4).

**Table 4:** Association between various factors with daytime sleepiness among healthcare staff ESS: Epworth daytime sleepiness scale.

Clinico-social factors		ESS		p-value
		No daytime sleepiness	Daytime sleepiness present	
Gender	Male	48 (40.3%)	71 (59.7%)	0.03
	Female	21 (25.6%)	61 (74.4%)	
Occupation	Junior residents	49 (45.8%)	58 (54.2%)	0.001
	Consultants	08 (32.0%)	17 (68.0%)	
	Nursing staff	12 (17.4%)	57 (82.6%)	
Marital status	Married	21 (31.3%)	46 (68.7%)	0.32
	Unmarried	48 (35.8%)	86 (64.2%)	
How many times did you contract COVID?	Only once	28 (29.2%)	68 (70.8%)	0.8
	Twice	17 (47.2%)	19 (52.8%)	
	More than 2 times	24 (34.8%)	45 (65.2%)	
Vaccination status	First dose	4 (100.0%)	00 (0.0%)	0.01
	Second dose	34 (29.8%)	80 (70.2%)	
	Precautionary dose	31 (37.3%)	52 (62.7%)	
How many hours do you work in the hospital?	Less than 8 hours	06 (9.2%)	59 (90.8%)	0.01
	More than 8 hours	63 (46.3%)	73 (53.7%)	
Are your shifts regular?	Yes	54 (31.6%)	117 (68.4%)	0.04
	No	15 (50.0%)	15 (50.0%)	
Do you smoke tobacco daily?	Yes	8 (29.6%)	19 (70.4%)	0.3
	No	61 (35.1%)	113 (64.9%)	
Do you consume alcohol daily?	Yes	08 (28.6%)	20 (71.4%)	0.3
	No	61 (35.3%)	112 (64.7%)	
Have you been admitted to the hospital for COVID-19 infection after vaccination?	Yes	21 (37.5%)	35 (62.5%)	0.3
	No	48 (33.1%)	97 (66.9%)	

Male HCWs had lesser odds of 0.51 (0.25-1.06) for daytime sleepiness, which was statistically significant. Junior resident HCWs had lesser odds of 0.34 (0.15-0.93). Duration of work more than eight hours had higher odds of 6.6 (2.4-17.7) with a p-value less than 0.01. Factors like smoking tobacco daily and alcohol consumption weekly had higher odds; however, it was not statistically significant (Table 5).

**Table 5:** Binary logistic regression analysis of daytime sleepiness with various factors HCWs: healthcare workers.

	B	S.E.	p-value	Odds ratio	95% confidence interval	
					Lower	Upper
Male HCWs	-0.656	0.367	0.04	0.519	0.253	1.066
Junior residents	-0.984	0.467	0.03	0.374	0.150	0.933
Consultants	-0.970	0.638	0.12	0.379	0.108	1.324
Married HCWs	-0.143	0.425	0.73	0.867	0.377	1.994
Contracted COVID infection once	0.548	0.405	0.17	1.729	0.782	3.823
Contracted COVID infection twice	-0.044	0.511	0.93	0.957	0.351	2.606
Duration of work more than 8 hours	1.888	0.505	0.0001	6.606	2.453	17.790
Smoke tobacco daily	0.040	0.546	0.94	1.041	0.357	3.036
Consume alcohol weekly	0.729	0.508	0.15	2.073	0.766	5.611
Admitted to the hospital for COVID-19 infection	-0.137	0.415	0.74	0.872	0.387	1.965

## Discussion

The present study is a cross-sectional study carried out for a period of one year to assess QoL and daytime sleepiness among younger HCWs who have been infected with COVID at least once. Out of 201 FLHCWs who completed the interview, the majority were male, junior residents, unmarried, and workers who had contracted infection only once. The majority said they followed regular shifts. After assessing QoL, male HCWs had higher scores in the psychological, social relationships, and environmental domains. Consultants had higher scores in all domains. Married HCWs had higher scores in the physical, psychological, and social relationship domains. HCWs who followed regular shifts had higher scores in the physical health and environmental domain. On assessing daytime sleepiness, female HCWs, nursing staff, FLHCWs working less than eight hours a day, and those HCWs who followed regular shifts, there was a statistically significant association between daytime sleepiness. However, binary logistic regression analysis showed doctors working for less than eight hours had higher odds for daytime sleepiness as the only significant factor.

The COVID-19 pandemic has given a newer dimension of hospital care and working challenge for HCWs. Longer working schedules, longer isolation time post-COVID infection, less experienced staff being the frontrunner in patient care, and no proper institutional strategies to handle such stressful situation has been an absolute necessity. The present study showed that males, consultants, and married workers had better QoL in various domains similar to various other studies. A study done by Buselli et al. showed that working in the frontline or being a COVID-treating physician positively impacts the QoL tremendously and younger physicians and younger nursing staff are at more risk due to lack of experience in hospital care while handling traumatic situations like the COVID pandemic. Females had poorer QoL compared to males, attributed to greater empathy for trauma and lower tolerance to negative emotions than men [19]. A study done in Serbia among healthcare professionals also showed the highly perilous nature of the COVID pandemic on HCWs displaying psychological issues followed by lower health-related QoL. It was observed among female healthcare professional nurses, married health workers, and health workers having children, working on the frontline at temporary COVID hospitals dealing with positive patients [20]. A study done in Indonesia has shown that HCWs working in COVID hospitals had lower QoL attributed to poor mental health support, dehumanizing experience while treating COVID cases, lesser social health assistance due to the very nature of the virus of being very contagious, and fear of infecting social circle resulting in abstaining from any friends or relatives or family members being considerable causes for their impaired QOL [21]. A study done by Rashid et al. in Bangladesh among HCWs infected with COVID showed that post-COVID QoL was affected by various demographic and socioeconomic determinants, including their age, gender, education, and monthly salary. One or more areas of QoL were significantly impacted when disease severity and the degree of comorbidities were considered [22]. A study done in China among younger high-risk HCWs showed that QoL was significantly affected among HCWs working as frontline workers [23].

The present study showed that moderate excessive daytime sleep and severe excessive daytime sleep were very common among COVID-infected frontline HCWs. Terms like “coronasomnia” or “COVID-somnia” are brought forward to encompass the cluster of sleep symptoms like dysfunction, insomnia, disrupted sleep continuity, changes in the sleep-wake cycle, feelings of non-restorative sleep, and the decreased sleep quality or the psychosocial impact on the daily living psychological sequel of contracting COVID-19 infection. There are shreds of evidence suggesting that a considerable proportion of COVID-infected HCWs have been experiencing sleep disturbances significantly higher than the general population [24,25]. A study done in Israel among HCWs who treat COVID cases has shown that sleep disturbances were very common and prompted the HCWs to maladaptive coping like increased tobacco smoking [26]. A cohort study done in Iran also showed that the COVID pandemic had a tremendous impact on the sleep and QoL of HCWs [27]. Sleep is a key indicator for healthy life and is directly related to a better QoL, and the COVID pandemic had robbed HCWs. During the second wave of COVID-19, India witnessed the highest number of mortalities and morbidities related to COVID. Indian HCWs worked in high-pressure and stressful environments, due to which those working in COVID-19-designated hospitals reported clinically significant depression and poor sleep [28]. A study done in India has also shown similar findings where sleep-deprived HCWs working during this pandemic have been pushed for stress, mental health illness, and non-adaptive coping strategies [29,30]. A study done in Italy among healthcare professionals attributed sleep disturbances to psychological stress and women were at more risk. The study also stressed that better coping strategies must be adopted by healthcare professionals and healthcare institutes must reinforce them so that there are no long time mental health consequences after COVID infection among HCWs [31]. The preliminary research shows the possible reasons why repeated infections can alter the sleep cycle. It is said that COVID virus alters circadian rhythm because of the pro-inflammatory response of this infectious allergen, impaired immunological memory of interleukin, and cytokines leading to anti-oxidant imbalance. This altered molecular level leads to oxidative stress and circadian rhythm misalignment. HCWs at the frontline are especially at risk of developing circadian rhythm problems due to changes in daily routine and an irregular sleep-wake schedule [32]. Vaccination can protect our immune system from such flared-up reactions but repeated infections, high workload, and increased stress can be detrimental and can damage circadian rhythm [33]. Younger HCWs should be educated to maintain sleep hygiene. Sleep hygiene intervention should be one initiative the working hospitals must take as a priority.

The present study has shown that even after various COVID vaccination doses received by the HCWs, sleep and QoL have still been impaired among infected younger healthcare staff. Standard validated tools were used to assess sleep and QoL among HCWs. Very few research have been published regarding younger HCWs.

Limitations of the study are a smaller sample size and the study being carried out among two health centers of the same geographical location. The multi-centric study would have added more evidence. The cause-and-effect relationship between COVID with impaired sleep and QoL cannot be established in the present study. The present study only studied daytime sleepiness; however, sleep quality assessment would have added better dimensions for sleep. The present study cannot establish the temporal association of COVID infection per se has affected their QoL and sleep.

## Conclusions

The present study showed that younger HCWs working during this pandemic have been infected with COVID and post-COVID effects have been witnessed among them. QoL and sleep are commonly affected along with various other symptoms. The present study included younger HCWs aged less than 30 years and working in COVID isolation wards, outpatient wards, and inpatient wards. Male HCWs, married workers, consultants, and health workers who followed regular shifts had a better QoL in various domains. Gender and occupation had a significant association with daytime sleepiness. Repeated infections, prolonged working schedules, and lack of experience to tackle this stressful situation have been major factors playing a plausible causative role. All healthcare administrators should design and sketch an outline of pandemic disaster preparedness and implement programs that reinforce HCWs during crisis events like this COVID pandemic. All efforts must be made in making sure these interventions are pragmatic, flexible, and responsive to unique health system pressures as modified by individual needs. Educating sleep hygiene among HCWs must be taken into account, as this intervention can have benefits, especially in dealing with infectious diseases. A bottom-up approach with young FLHCWs as one of the key stakeholders in policy decisions should also be considered. Younger FLHCWs predominantly form a treating team for any highly infectious and contagious illness. All efforts must be made to shield them from the direct consequences of the pandemic on physical health because of infection and indirect consequences on mental health.
